# Impact of Infant-Polysomnography Studies on Discharge Management and Outcomes: A 5 Year Experience from a Tertiary Care Unit

**DOI:** 10.4172/2167-0897.1000257

**Published:** 2017-05-31

**Authors:** Ahmed Fageer Osman, Biju Thomas, Nakul Singh, Marc Collin, Prem Singh Shekhawat

**Affiliations:** Department of Pediatrics, Division of Neonatology, Case Western Reserve University, MetroHealth Medical Center, Cleveland, USA

**Keywords:** Polysomnography, Infant apnea, Acute life threatening event, Home monitoring

## Abstract

**Objective:**

To evaluate the impact of infant-polysomnography studies performed in the NICU on management and outcomes.

**Study design:**

Retrospective study to collect demographics and data on infant-polysomnography studies between Jan 2010 to Dec 2014.

**Results:**

110 premature neonates had polysomnography study performed at 36.9 ± 2.5 weeks post menstrual age. Almost all the studies were read as abnormal and 95% of the studied infants were discharged home on a cardiorespiratory monitor. 20% of the subjects had apnea >20 s, 18% had apnea of 15–20 s and 50% of infants had apnea of 10–15 s. 24.5% infants were discharged home on caffeine, 28% on metoclopramide and 24% on antacids. There were 11 readmissions for apparent life threatening events with no until 6 month-corrected age. There was no association between polysomnography results and readmission. There was a decline in polysomnography studies performed each year.

**Conclusion:**

Cardiorespiratory monitoring, medications and polysomnography studies do not predict outcomes.

## Introduction

Infant-polysomnography studies are commonly performed in the Neonatal Intensive Care Units (NICU) on premature infants around the time of discharge to assess the risk of central and peripheral apneas, bradycardia, desaturations and gastroesophageal reflux events, in an attempt to prevent apparent life threatening events (ALTE). Apnea of prematurity (AOP) is defined as a cessation of breathing that lasts for at least 20 s or at least 10 s followed by bradycardia and hypoxemia witnessed in infants <37 weeks gestation [1–5]. The incidence of recurrent apnea is inversely related to gestational age at birth. Almost all infants born at or less than 28 weeks of gestation will experience some degree of apnea [6]. Regular, repeated cycles of breathing lasting 10–15 s each with apneic pauses of 5–10 s called periodic breathing is quite common in premature infants [7,8]. Neonatal apnea is frequently attributed to gastroesophageal reflux (GER) and medications for GER are commonly prescribed to these infants [9].

Apneas, bradycardias and desaturations (ABD) are major factors in prolonging hospitalization and delaying discharge among premature neonates. Increase in frequency of ABD events may also be a sign of acute pathological process like late-onset sepsis, necrotizing enterocolitis (NEC) etc. and usually leads to a diagnostic workup in such infants [10]. There is concern that these events may lead to a number of adverse outcomes in the long term, such as Retinopathy of Prematurity (ROP), poor neurodevelopmental outcome, increased risk of apparent life threatening events and sudden infant death syndrome (SIDS) [11]. It has been postulated that chronic intermittent hypoxia increases free radical production and may contribute to the immediate and long-term co-morbidities in this population. Intermittent hypoxia in extremely premature infants has been found to be associated with late death or disability [12].

Polysomnography studies have been utilized to assess potential mechanisms of these apneic events so that an intervention may be provided to such fragile neonates [13]. However, there is paucity of data regarding the value of these studies and their impact on short and long term outcomes; hence there is wide variation in practice among NICU’s. Limiting the practice of performing polysomnography studies for premature infants was among the five targets of American Academy of Pediatrics’’ “Choose Wisely in Newborn Medicine campaign” [13]. In view of this recent campaign, we audited our practice to study the value of polysomnography studies performed in our NICU by analyzing the short and long term outcomes among our NICU graduates.

## Methods

### Subjects and study design

Using a retrospective cohort study design, we examined the medical records of all premature neonates (<37 weeks gestation), admitted between January 1^st^, 2010 and December 31^st^, 2014. Our NICU is a tertiary care 49 bed level IIIB unit with L&D service at MetroHealth Medical Center (MHMC), which is affiliated with Case Western Reserve University, Cleveland, OH. The study subjects were identified from our neonatal outcomes database using the terms apnea, bradycardia, desaturation, sleep study and acute life threatening event (ALTE). We then identified subjects who underwent polysomnography studies prior to their discharge from the NICU. The study was approved by Case Western Reserve University Institutional Review Board (IRB). A waiver was granted for a formal informed consent and HIPAA authorization on these subjects.

Polysomnography studies were performed using the Respironics equipment (Murrysville, PA) and reported by a single pediatric pulmonologist at MHMC. All the studies were performed while the subjects were inpatient in the NICU. Each study monitored and recorded the following channels: nasal airflow by thermistor, oxygen saturation by pulse oximeter, heart rate by precordial chest sensor, and chest wall effort/motion by impedance.

### Inclusion and exclusion criteria

Infant born at less than 37 weeks of gestation (determined by obstetric data) and born between January 1^st^, 2010 to December 31^st^, 2014 were included if they underwent polysomnography study during hospitalization in the NICU. Exclusion Criterion was the presence of a major congenital anomaly.

We collected demographic data, sleep study results, discharge medications, discharge equipment, re-admissions due to ALTE and mortality data until 6 month-corrected age. Study data was collected and managed using REDCap (Research Electronic Data Capture) electronic data capture tools hosted at MHMC [14].

### Statistical Methods

Data was collected in a secure REDCap database [14] which was later exported to Microsoft Excel spreadsheet for initial analysis and then to open source software “R” prog, version 3.3.0 (https://www.r-project.org/) for elaborate statistical analysis. The continuous variables were described as mean, standard deviation, median, interquartile range (IQR) and minimum and maximum values. Differences in continuous variables were tested with Student’s ‘T’ test. Three quantities were calculated from each infant’s sleep study data: the number of apneic episodes lasting greater than 10 s, i.e., average number of apneic episodes per study period of 6 h. Desaturation was defined as O_2_ saturation <90% for percent of study time. Since it was of clinical interest to estimate the relationship between frequency of apnea, desaturations (%) and length of longest apneic episode and three well-known predictors of infant outcome: gestational age at birth, birth weight and age at study so each of the three sleep study variables were set as dependent variables and each of the three predictors were set as the independent variables in a series of linear regressions (a total of nine regressions). A p value <0.05 was considered statistically significant.

## Results

### Demographic and outcomes characteristics

**Figure 1:**
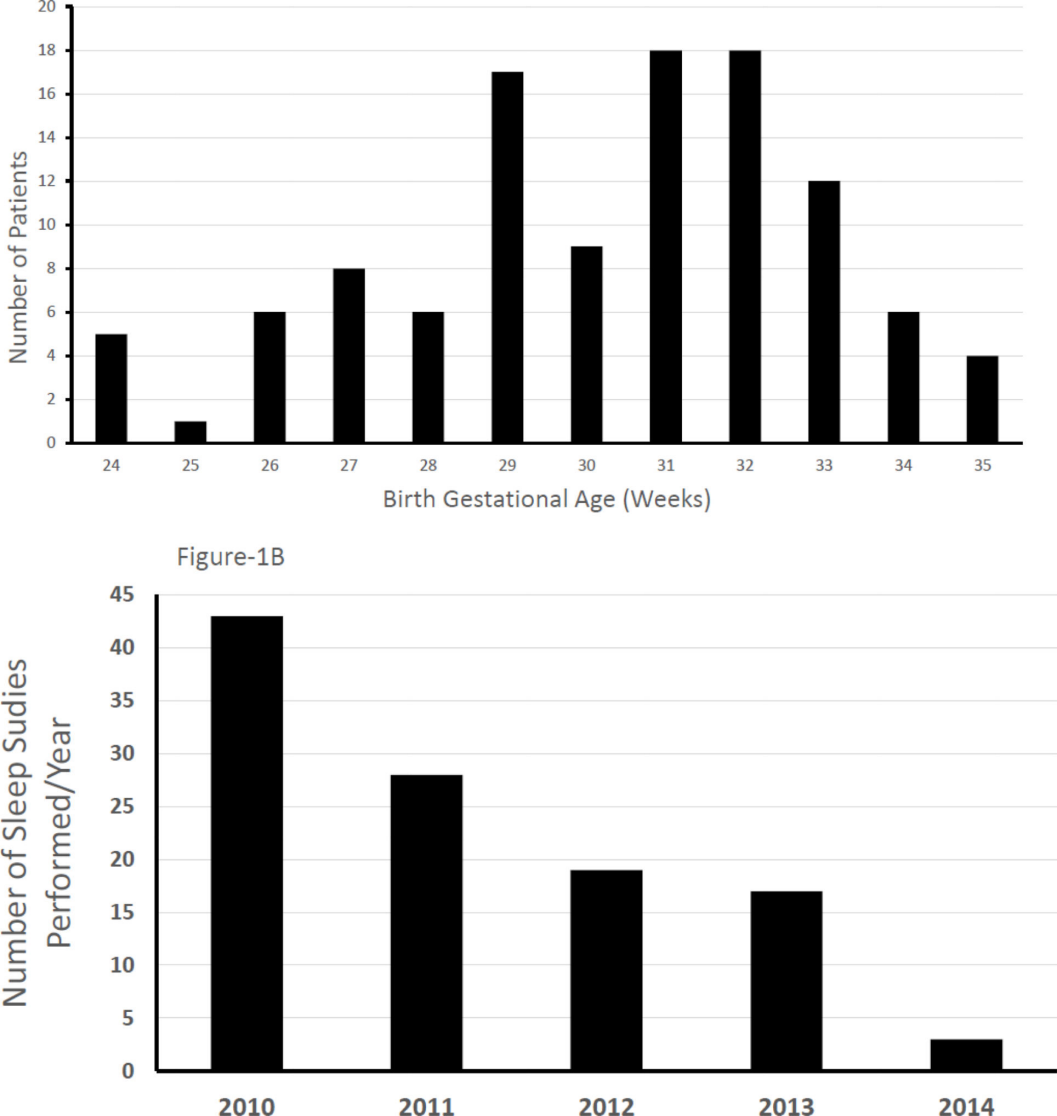
Distribution and use of polysomnography studies between 2010–2014 across various gestational age groups.

We identified 110 premature neonates who met the study inclusion criteria and their distribution over the study period is as shown in Figure 1A. Their birth gestation ranged from 24 to 35 weeks, with a mean ± SD of 30.2 ± 2.7 weeks and their birth weight ranged from 436–849 g with a mean ± SD of 1425 ± 453 g. The overall distribution of the subjects was 44% Caucasians, 44% African American and 11% Hispanic. Forty-seven percent were males and 53% females. The number of sleep studies performed each year showed a pattern of steady decline as shown in Figure 1B.

The postnatal age at the time of polysomnography study ranged from 1 week to 19 weeks, with a mean of 6.7 weeks and a median of 6 weeks. The mean postmenstrual age at of study was 36.9 weeks with a median of 36 weeks. The median postmenstrual age at discharge was 37 weeks (IQR: 36–39 weeks), median weight at discharge was 2623 g (IQR: 2319–3079 g).

The median postmenstrual age at hospital discharge was 37 weeks (IQR: 36–39 weeks), while the median weight was 2623 gs (IQR: 2319–3079). All infants were on full oral feeding at discharge, 81% were discharged on 22 Kcal/Oz cow’s milk based formula, 14.5% were on breast milk, with or without added formula and 7% infants were prescribed an elemental formula.

### Polysomnography study results

The duration of polysomnography studies was kept constant around 360 min and the median duration of actual sleep during the study was 220 min (62%) (IQR: 210–238 min). The patterns of central apneas in the subjects were as follows: 13 infants (12%) did not have any central apnea (≥ 10 s), 55 infants (50%) had maximal central apneas between 10–15 s, 20 infants (18%) had maximal central apneas of 15–20 s and 22 infants (20%) had maximal apneas lasting >20 s. The longest central apnea was 49 s, with median of 15 s (IQR: 12–18 s).

The percentage of time the patients had oxygen saturation below 90% ranged from 0 to 27%, with a median of 1.5 % (IQR 1.0–4.3%). Median duration of periodic breathing was 8.5% of the time (IQR: 0.8–20.7%). 1 subject was diagnosed with obstructive apnea where apnea duration was between 10–15 s. 8 subjects were diagnosed with mixed apneas where maximal apneas of 3 subjects were between 10–20 s, while the maximal apneas of the remaining 5 subjects were >20 s. All the studies were interpreted by our pediatric pulmonologist as abnormal. Almost all subjects were recommended to have cardiopulmonary (CR) monitoring after discharge home till their ABD events improved on follow up.

11 (10%) infants experienced an acute life threatening event (ALTE) and required re-admission to the hospital by 6 months corrected age. There was no association between ALTE related hospital re-admission and gestational age at birth, birth weight, age when polysomnography study was performed, or duration of time infant had oxygen saturation <90% during the study as shown in Table 1. Unpredictably infants re-admitted for ALTE actually had less number of apneic episodes compared to those who were not readmitted (p=0.04).

Table 2 shows the composite results of the regressions analysis of various sleep study variables with the infant outcome predictors of gestational age at birth, birth weight, and age at study. The number of apneic episodes increased with the gestational age at birth and with birth weight, but decreased with increasing age at study. Specifically, for each week increase in gestational age at birth, there was an average about 1.81 more apneic episodes per day (p=0.04); a 100 g increase in birth weight was associated with 1.62 more apneic episodes per day (p=<0.01); each additional week of age at time of the study was associated with 1.44 fewer apneic episodes per day. Gestational age at birth, birth weight, and age at study were not associated with percent of time desaturation was noted. Each 100 g increase in birth weight was associated with a 0.39 s increase in length of longest central apneic episode (p=0.01) and each week increase in age at study was associated with a 0.36 s decrease, but gestational age was not associated with length of longest central apneic episode. The postnatal age seems to the major determinant of neurological maturity of each infant which leads to lesser number of apnea and desaturation episodes and improved outcomes. Thus an extreme preterm infant born at 24 weeks gestation who is corrected to 35 weeks corrected gestational age is less likely to have apnea and desaturations compared to 1 week old 34 week gestation infant.

### Discharge and follow up patterns

Recommendations made by our pulmonologists after conclusion of polysomnography studies are summarized in Table 3. Based on these recommendations workup for GER, dysphagia or both was performed in several infants. Adequacy of oxygen saturation and need for home oxygen was recommended to be assessed before discharge home in nearly 15% of infants. CR monitoring was initiated on 105 infants (95%) at discharge, while 27 infants (24.5%) were discharged on caffeine, 31 (28%) were on metoclopramide and 26 (24%) on antacid mediation. CR monitor was discontinued at a median postmenstrual age of 46 weeks. The duration of use of home CR monitor and medications following discharge is outlined in Table 4. 103 infants (94%) were known to be alive at 6 months corrected age, while 7 infants (6%) were lost to follow up. No infant was known to have died by 6 months of corrected age.

## Discussion

Our study from a single tertiary care center show that majority of infants who underwent polysomnography studies were born between 29–33 weeks of gestation, while the number of extremely premature infants were small in comparison. Infants in our study population were asleep for about 60% of the study time which was comparable to other investigators [15]. The median and mean postmenstrual age when the polysomnography studies were performed was constant at 36 weeks, the time when apnea of prematurity is normally expected to improve and the infants are close to being discharged home. Thus it seems postnatal age was a major determinant of neurological maturation of these infants, which led to improvement of their ABD events and clinicians were comfortable discharging them home without a CR monitor.

The majority of reported apneas were central in origin, in contrast to previous reports of mixed apnea being the most prevalent type of apnea [1,10,16]. Half of our subjects spent 8.5% or more of the study time in periodic breathing which was in line with other recently reported value of about 6% for infants <35 weeks of gestation [6]. Our review of polysomnography studies surprised us that all the studies were reported as abnormal for a variety of reasons, which reflects absence of standardization in reporting these studies for each population [17,18]. Likewise use of cardiorespiratory monitoring was recommended for almost all of the study subjects, this practice was not supported by any of the consensus statement in the literature or any individual reports. It has been reported that premature infants at higher risk of SIDS, there is no evidence to suggest that home CR monitoring helps prevent SIDS [19]. American Academy of Pediatrics’ clinical report states that CR monitor may be prescribed for some preterm infants with an unusually prolonged course of recurrent extreme apnea, and such monitoring should be discontinued in most infants after 43 weeks. In contrast the time of discontinuation of CR monitor in our subjects was 46 weeks and thus not consistent with recent recommendation by the AAP [4,20]. Thus there is a need to standardize the reporting of polysomnography studies and clear indications are needed for use of home CR monitoring since they both add to the cost of patient care with no clear demonstrable benefit to the patients.

Almost 28% of our subjects were prescribed either a H_2_ blocking agents or a prokinetic agents like metoclopramide or both. There was a prolonged use of these medications in some of our subjects in spite of a ‘black box warning’ by the FDA for metoclopramide since it leads to an increased risk of tardive dyskinesia [21,22]. GER medications have been shown to have several side effects like sepsis, necrotizing enterocolitis besides tardive dyskinesia and several studies have failed to show a correlation between use of these medications and apnea, bradycardia or respiratory symptoms typically thought to be due to GER [23].

Our study has several limitations in that, it is a single center observational study and all our polysomnography studies were read by a single clinicians which may introduce some bias but our data show that we are in line with other published literature and our conclusions are consistent with current AAP recommendations [19–21,24,25].

## Conclusion

In conclusion we can say that polysomnography studies in our institution like other centers have recently fallen out of favor since most clinicians feel that these studies do not guide those regarding therapeutic options and cannot help prognosticate long-term outcomes. Our data indicate that postnatal age is a major determining factor for maturity of nervous system rather than gestational age at birth and findings on sleep studies do not predict the occurrence of ALTE or death by 6 months corrected age. Thus use of polysomnography studies, home CR monitoring and GER medications should be limited to some of the most unstable infants.

## Figures and Tables

**Table 1:** Demographic data and infant-polysomnography study characteristics of infants readmitted to the hospital with ALTE versus those who were not.

Demographic data/ polysomnography study’s findings	Infant not readmitted to the hospital	Re-admitted with ALTE	P value
Birth Gestational Age (Weeks)	30.2 ±2.8	30.5 ± 2.2	0.7
Birth Weight (Grams, g)	1420 ± 456	1471 ±442.3	0.72
Age at study	6.7 ± 3.9	6.5 ± 4.8	0.91
Number of apneic episodes detected on polysomnography	6 ± 4	4 ± 6	0.04
Desaturation (% of time)	1.0 ± 1.3	1.1 ± 1.1	0.83
Longest Central Apnea (Seconds, s)	16.1 ± 7.2	15.4 ± 3.4	0.58

**Table 2:** Relationship between number and duration of apneic episodes and desaturation events and gestational age at birth, birth weight and age at study using regression analysis.

	Estimate	SEM	P value	R2
**Number apneic episodes/day**				
Gestational age at birth	181.00%	85.00%	0.04	0.04
Birth Weight (100 g)	1.62	0.5	0.001	0.09
Age at study (Weeks)	−1.44	0.58	0.02	0.05
**Longest central apnea**				
Gestational age at birth	0.45	0.24	0.07	0.03
Birth Weight (100 g)	0.39	0.14	0.01	0.07
Age at study (Weeks)	−0.36	0.16	0.03	0.04
**% time infant had desaturation**				
Gestational age at birth	0.04	0.17	0.83	0
Birth Weight (100 g)	0.09	0.1	0.39	0.01
Age at study (Weeks)	0.01	0.12	0.96	0

**Table 3:** Recommendations other than use of CR monitor made by pediatric pulmonologist based on polysomnography study findings.

Recommendation	Number of the patients (%)
Assessment for adequacy of oxygen saturation	17 (15%)
Clinical Assessment/Evaluation	9 (8%)
Assessment for GER/GERD	8 (7%)
Assessment for GERD and dysphagia	6 (5%)
Assessment for dysphagia	5 (4.5%)
Assessment for adequacy of saturation+GERD+Dysphagia	2 (2%)
Follow up sleep study	2 (2%)
Assessment for adequacy of saturation+Dysphagia	1 (1%)

**Table 4:** Use of cardiorespiratory monitor and medications.

Intervention	No. of patients (%)	Median duration of use as outpatient in weeks (IQR)
Discharged home on CR monitor	105 (95%)	9 (6–11)
Discharged home on caffeine	27 (24.5%)	5.5 (4–8.25)
Discharged home on metoclopramide	31 (28%)	15 (9.25–21.72)
Discharged home on antacid mediations	26 (24)	14 (10.5–23.5)
